# Extending Coronectomy Indications to Third Molars with Taurodontism to Prevent Paresthesia and Mandible Fracture

**DOI:** 10.1155/2018/2067350

**Published:** 2018-04-01

**Authors:** Polianne Alves Mendes, Isabela Moreira Neiva, Cláudia Borges Brasileiro, Ana Cristina Rodrigues Antunes Souza, Leandro Napier Souza

**Affiliations:** ^1^Department of Oral and Maxillofacial Surgery, School of Dentistry, Universidade Federal de Minas Gerais, Belo Horizonte, MG, Brazil; ^2^Department of Dentistry, Centro Universitário Newton Paiva, Belo Horizonte, MG, Brazil

## Abstract

Taurodontism is considered a dental anomaly responsible for a morphoanatomical change in the shape of the tooth in which the roots are reduced in size but the body of the tooth is enlarged and bulky. The aim of this paper is to present a case of a 25-year-old female patient with taurodontism of mandibular partially erupted third molars, presenting a high risk of angle fracture and paresthesia in case of their removal, treated by means of coronectomy. The postoperative period was uneventful and the patient remained in follow-up for 12 months. In conclusion, the identification of third molars with higher risk of complications related to their extractions is the key to consider conservative measures to avoid problems. Coronectomy is a relatively simple technique that should be taken into account when considering bulky, deeply located third molars with a high risk of paresthesia or mandibular fracture, even in presence of taurodontism.

## 1. Introduction

Taurodontism is characterized by an enlarged pulp chamber with apical displacement of pulp floor and absence of cementoenamel junction constriction diagnosed by radiographic examination. This dental anomaly is caused by failure on invagination of the Hertwig epithelial sheet diaphragm at the appropriate horizontal level, leading to changes in tooth shape [1–3]. Fracture of the mandible during extraction is a rare but underestimated complication, and most cases are associated with the removal of voluminous or deeply impacted third molars [4, 5]. Coronectomy appears as a preventive surgical technique and represents an excellent alternative to conventional extraction, generating less morbidity in surrounding tissues and preventing mandibular fractures and paresthesia [6–8].

## 2. Case Presentation

**Figure 1:**
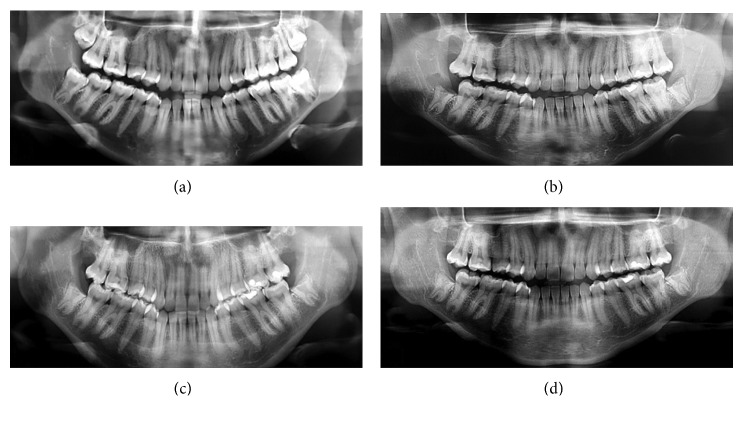
(a) Initial panoramic radiograph and (b–d) follow-up with 7 days, 6, and 12 months, respectively.

A 25-year-old female patient presented to the Oral and Maxillofacial Surgery Service complaining of mandibular third molars with need for removal. She reported that she had sought another service, in which the extraction was indicated in a hospital, under general anesthesia, with posterior internal fixation of the mandible with plates and screws due to the risk of mandibular fracture. Panoramic radiograph revealed partially erupted third molars compatible with taurodontism extending up to the base of the mandible (Figure 1). Medical history was not relevant. Computed tomography was performed to identify related anatomical structures revealing close relation to the mandibular canal in both sides. So, considering the risk of mandibular fracture and paresthesia in both sides, coronectomy was proposed to both teeth under local anesthesia. After patient's consent, intraoral approaches consisting of conventional accesses for third molars were performed. Only the crowns were removed through bur sectioning, under copious saline irrigation, at the level of cementoenamel junction, keeping the roots intact and thus avoiding dislocation and force transmission. Consequently, damage to the inferior alveolar nerve and occurrence of mandibular fracture were prevented. A diamond spherical drill was used to regularize the surface of the remaining roots. Primary closure was made with 5.0 nylon sutures and removed after 7 days. Surgery and postoperative period were uneventful, and control radiographs were done at 7 days, 3, 6, and 12 months. Roots migration occurred in both sides (Figure 1). After 1-year follow-up no complications were observed with complete healing on both sides.

## 3. Discussion

Diagnosis of taurodontism is mainly based on features that are particularly best seen on radiograph, as in the case presented herein. Although it appears more frequently as an isolated anomaly, mainly in permanent molar teeth, its association with various syndromes and abnormalities has also been reported [1, 2]. Nevertheless, in the case reported, no association with syndromes was present.

Extraction of teeth presenting taurodontism is usually complicated because of the change in furcation and volume of the tooth [3] and could lead to a risk of mandibular fracture, especially in cases with tooth length extending to the basilar as in the current case. Iatrogenic mandibular fracture associated with tooth removal can be the most serious complication and can occur immediately during the procedure or later in the first 4 weeks, being mostly associated with removal of third molars [4]. The danger of an immediate mandibular fracture can be avoided by appropriate instrumentation and by abstaining from excessive bone strength [5].

Coronectomy is reported as a less traumatic treatment alternative, in which the tooth should be sectioned, removing only the crown and maintaining the roots and minimizing the extent of bone removal and the force caused by the instrumentation. The success of coronectomy depends on permanence of root fragments, which must be successfully retained, together with the subsequent formation of bone and cement on the roots. For this, all enamel must be removed. This procedure can be performed safely on impacted third molars regardless of their classification, minimizing the amount of bone removed and allowing reduction of force applied during dislocation [6, 7]. However, clinical and radiographic follow-up should begin immediately and maintained for 12 months [8]. In the case presented, coronectomy was done in both lower third molars with taurodontism without complications during 1-year follow-up. So, considering the risks of surgical removal of third molars with taurodontism, coronectomy should be taken into account as the treatment choice.
